# Prefrontal activity and heart rate variability during cognitive tasks may show different changes in young and older adults with and without mild cognitive impairment

**DOI:** 10.3389/fnagi.2024.1392304

**Published:** 2024-05-28

**Authors:** Pei-Hsin Ku, Yea-Ru Yang, Nai-Chen Yeh, Pei-Yun Li, Chia-Feng Lu, Ray-Yau Wang

**Affiliations:** ^1^Department of Physical Therapy and Assistive Technology, National Yang Ming Chiao Tung University, Taipei, Taiwan; ^2^Department of Biomedical Imaging and Radiological Sciences, National Yang Ming Chiao Tung University, Taipei, Taiwan

**Keywords:** aging, prefrontal cortex, heart rate variability, cognitive function, executive function, mild cognitive impairment

## Abstract

**Background:**

Age-related decline in cognitive function is often linked to changed prefrontal cortex (PFC) activity and heart rate variability (HRV). Mild cognitive impairment (MCI), a transitional stage between normal aging and dementia, might have further degeneration beyond aging. This study aimed to investigate the differences between young and older adults with or without MCI in cognitive functions, task-induced PFC activation and HRV changes.

**Methods:**

Thirty-one healthy young adults (YA), 44 older adults (OA), and 28 older adults with MCI (OA-MCI) were enrolled and compared in this cross-sectional study. Each participant received a one-time assessment including cognitive and executive functions, as well as the simultaneous recording of PFC activity and HRV during a cognitive task paradigm.

**Results:**

We observed age-related decrease in global cognitive functions, executive functions, HRV, and increase in PFC activity. The MCI further deteriorated the global cognitive and executive performances, but not the HRV or the prefrontal activation.

**Conclusion:**

Older people showed lower performances in general cognitive function and executive function, compensatory increase of PFC activity, and reduced HRV. Older people with MCI had further deterioration in cognitive performance, but not in PFC activation and HRV.

## 1 Introduction

As the proportion and absolute number of older people increase dramatically worldwide, aging has become a critical issue. When we age, the brain degenerates structurally and functionally. Prefrontal cortex (PFC), playing essential role in top-down modulation of cognitive function (Funahashi and Andreau, 2013), is frequently reported to have the most substantial age-related changes. PFC degeneration correlates with cognitive decline and increases risks of neurodegenerative diseases in older adults (Salat et al., 2001; Xu et al., 2019). Structural decay and functional hyperactivation in PFC were found to be associated with poor performance in executive function and memory in older adults (Heuninckx et al., 2005, 2008; Deary et al., 2006; Emery et al., 2008; Davis et al., 2009).

A growing number of studies presented the importance of autonomic nervous system (ANS) in aging and cognitive processing in addition to PFC. The neurovisceral integration model proposed by Thayer et al. (2009) and Thayer and Lane (2009), has brought forward the interplay between cognitive processes and ANS. The integral component of the internal regulation from brain to visceromotor, neuroendocrine, and behavioral responses is expressed as the central autonomic network (CAN). This entity supports goal-oriented behaviors and adaptability (Benarroch, 1993; Thayer et al., 2009). The output of CAN directly links to heart rate variability (HRV), and therefore HRV is considered as an indicator of CNS-ANS integration. In particular, HRV is sensitive to the neural flexibility in response to situational requirements. The aging ANS usually shows increased sympathetic activity and the relative decrease in parasympathetic activity, or vagal tone. In geriatric communities, ANS impacts cognition and potentially predicts memory loss independent from genetic risk factors (Shah et al., 2011; Saint Martin et al., 2013). Some studies suggested that ANS dysregulation may contribute to determining successful/unsuccessful aging, as well as cognitive-related disease, such as Alzheimer’s disease or other dementia (Daulatzai, 2012; Vasudev et al., 2012).

The association between HRV and cognitive function has been reported in several studies. Johnsen et al. (2003) revealed that the group with low HRV required longer reaction time in Stroop test than high HRV group. Hansen et al. (2003) also reported that high HRV group not only showed less reaction time but also higher correctness in executive function task than low HRV group. A systematic review summarized the influence of HRV on cognitive function, pointing out the possibility of HRV serving as an early biomarker for cognitive impairment (Forte et al., 2019). Previous studies also suggested that cognitive task-related HRV changed in parallel with PFC activity. Gianaros et al. (2004) found the decrease of task-induced heart period and vagal tone were associated with decreased regional cerebral blood flow in medial PFC. Nikolin et al. (2017) demonstrated increased task-induced and resting high frequency rhythm, an index for vagal tone, by applying transcranial direct current stimulation on dorsal lateral PFC as compared to sham group.

Mild cognitive impairment (MCI), a transitional stage between normal aging and dementia, is the onset and evolution of cognitive impairments beyond those expected based on the age and education of the individual (Petersen et al., 2014). As MCI progresses, decrease in cortical thickness were observed in widespread areas, including bilateral prefrontal areas, leading to worse cognitive performance compared to healthy controls (Ye et al., 2014). Some studies reported people with MCI showed higher task-induced cerebral activation than age-matched healthy controls, and such activation positively correlated with task performance (Sperling, 2007; Liang et al., 2011; Clément et al., 2013). Others observed decreased brain activity in people with MCI in parallel with cognitive decline, as the capacity of brain resources has been reached (Holtzer and Izzetoglu, 2020; Yeung and Chan, 2020). In addition, the autonomic dysfunction, particularly the parasympathetic division, is common in people with cognitive impairment. People with MCI were 5.6 times more likely to have autonomic dysfunction than the controls (Collins et al., 2012; da Silva et al., 2018).

Although cognitive degeneration is a natural process in aging, whether the subtle cognitive decline in MCI compared to healthy older adults (OA) would make differences in broader cognitive assessments, as well as the cognitive task-induced brain activation and autonomic response was unknown. Therefore, our study aimed to investigate the differences between young and OA with or without MCI in cognitive functions, task-induced PFC activation and HRV changes. We hypothesized that young adults (YA) would have greater performance in cognitive assessments, lower PFC activation and higher HRV compared to OA. And OA-MCI would have the poorest cognitive performance, highest PFC activation, and lowest HRV among three groups.

## 2 Materials and methods

### 2.1 Participants

Adults from local communities in Taipei, Taiwan, were recruited between January 2019 to February 2022. The inclusion criteria for young adults were (1) age between 20 and 30 years, and (2) with Montreal cognitive assessment (MoCA) > 26. The inclusion criteria for older adults were (1) age ≥ 65 years old, and (2) with MoCA > 26. The inclusion criteria for participants with MCI were (1) age ≥ 65 years old, and (2) with MoCA < 26. The exclusion criteria were (1) diagnosis of neurological disorders, (2) MMSE < 24, (3) any current or previous cardiac conditions that might alter cardiac rhythms, including history of myocardial infarction, heart valve disease, prior cardiac surgery, heart failure, cardiomyopathy, and usage of cardiac-related medications, (4) diagnosis of autonomic dysfunction or dysautonomia, (5) uncontrolled hypertension or diabetes, (6) pregnancy. Comorbidities such as hypertension and diabetes were not excluded if participants were on regular medications and the blood pressure level or blood sugar level were within normal range. All participants were informed about the research procedures and signed a written consent form. The study was approved by the Institutional Review Board of National Yang Ming Chiao Tung University and registered prospectively as TCTR20181116001 at https://www.thaiclinicaltrials.org/.

### 2.2 Study design and measurements

This was a cross-sectional study. Participant received a one-time measurement, including cognitive performance, HRV and PFC activity during cognitive tasks. Characteristic data such as age, gender, educational level, height and weight were obtained at the beginning of the assessment. The general cognitive functions were measured with MMSE and MoCA and the executive function (EF) was measured by Trail Making Test (TMT) and Digit Span Test (DST). TMT test is one of the most widely used test for attention, sequencing and shifting ability of EF.

The TMT has 2 parts, A and B (Sánchez-Cubillo et al., 2009). Our team has documented that TMT-A and TMT-B (Chinese version) are feasible for Taiwanese elderly with high test-retest reliability (Wang et al., 2018). In TMT-A, participants were given a sheet of paper with number 1–25 and they were asked to draw lines to connect all the numbers in an ascending order. In Chinese version TMT-B, the English letters were replaced by 12 Chinese zodiac sign (rat, ox,…dog, and pig). Number 1–12 and the Chinese characters of the zodiac sign distributed on the paper. Participants were instructed to draw lines to connect numbers in an ascending order and zodiac signs in a set sequence, but the numbers and the zodiac signs must alternate (1-rat-2-ox,…12-pig). Participants should complete the “trails” as quickly as possible and the time needed to correctly connect all the dots was recorded as the test results.

The DST is a subtest in Wechsler Adult Intelligence Scale Revised and is commonly used for testing working memory of EF (Ruchinskas, 2019). The test is composed of forward span and backward span. Participants heard a sequence of number digits, and prompted to repeat the sequence of digits in the same order (forward span test) or in a reverse order (backward span test). If the participants correctly repeated the sequence, they moved on to the longer sequence. However, when the sequence was not repeated correctly, the test terminated immediately. The total score was the summation of how many sequences recalled, including forward and backward.

After the abovementioned cognitive measurements, participants underwent a cognitive task paradigm while the PFC brain activity was recorded by functional near-infrared spectroscopy (fNIRS) and the ANS activity was acquired by 3-lead electrocardiography (ECG) simultaneously.

#### 2.2.1 Cognitive task paradigm

The cognitive task paradigm was composed of three task conditions: Stroop Color Word Test (Scarpina and Tagini, 2017) with congruent condition (SCWT-c), SCWT with incongruent condition (SCWT-ic), and Category Fluency Test (CFT). These tasks were selected because they indicate the EF and memory which are the most frequently influenced during aging.

The SCWT was displayed by digital slides in Chinese characters. Participants were asked to read out the “character” in congruent condition (SCWT-c), where the color matched the character, and the “color” in incongruent (non-matched) condition (SCWT-ic), as quickly as possible. Both the congruent and incongruent tests lasted for 60 sec instead of 45 sec due to necessity of recording the brain activity and HRV simultaneously. SCWT-c reflects visuospatial processing speed and SCWT-ic measures inhibitory control as an important component of EF (Scarpina and Tagini, 2017). The CFT is frequently used for deciding semantic memory (Shao et al., 2014). The participants were asked to name as many kinds of animal, fruit or household items as possible within 60 sec.

**FIGURE 1:**
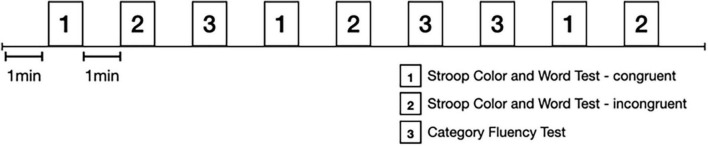
Testing paradigm.

Each task was performed in a 60-sec trial and repeated alternatively three times in a certain sequence with 60-sec rest between trials. The task sequence is displayed in Figure 1. The participants were instructed to remain quiet and relaxed during the resting period. The prefrontal activity and ANS activity were recorded continuously during the paradigm.

#### 2.2.2 Signals retrieval and data processing of prefrontal brain activity

**FIGURE 2:**
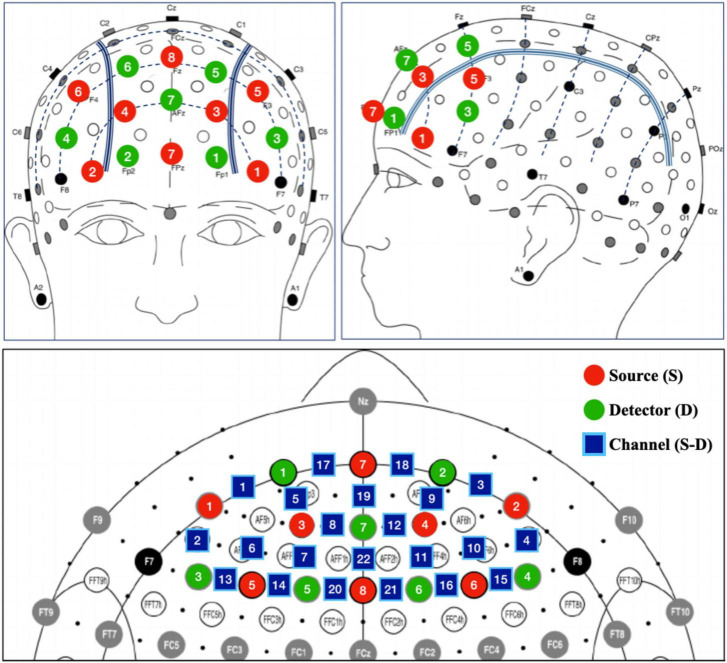
Placement of fNIRS probes. Detector 8 was used as referenced optode and thus not included in the prefrontal placement layout.

The prefrontal activity was recorded by a dual-wavelength (760 and 850 nm), multi-channel fNIRS imaging system (NIRSport2, NIRx Medizintechnik GmbH, Berlin, Germany). Sixteen optodes (8 LED light sources and 8 detectors) were placed on the scalp to collect the hemodynamics in prefrontal cortex with a head cap compatible with the international 10-5 system (Figure 2).

The signals were acquired with Aurora fNIRS, the default NIRSport 2 acquisition software, and then exported to be pre-processed using HOMER2 package. The calculation of coefficient of variation, concentrations of oxygenated hemoglobin (HbO), and statistical analysis were run with customized scripts created using MATLAB (2022b, Mathworks, Natick, MA, United States).

Channels with coefficient of variation (CV) > 10% were excluded to ensure the signal quality. The remaining signals were then filtered with band-pass filter. Low cutoff frequency was set at 0.008 Hz and high cutoff at 0.20 Hz to eliminate the influence of heartbeats, respirations, and low frequency drift. We subsequently applied wavelet filtering to correct motion artifacts. The preprocessed signals were further converted to the concentration in HbO using the modified Beer–Lambert law for each source-detector channel. A 5-s HBO level before each tasking trial was used as the baseline correction for the following 60-s tasking trial. The activity of each task was shown by the average of HbO over three trial repetitions.

#### 2.2.3 ANS activity

Heart rhythms were obtained by 3-lead ECG using the BIOPAC MP160 ECG data acquisition module (BIOPAC Systems, Inc., CA, USA). Electrodes were placed under each clavicle, and the third one on the lower left rib cage. The heart rhythms were recorded aligned with the task paradigms as the prefrontal activity measurements. An IIR high-pass filter with a cutoff frequency of 0.01 Hz was applied to the signals in order to eliminate the slow drift. The quantitative metrics were extracted using the built-in AcqKnowledge software and imported to Nevrokard-HRV Software (Slovenia) for R-peak identification and HRV indices transformation. R-R interval (RRI) is the time between two successive R-peak, which is the basic temporal unit used for further analysis. Two time-domain measurements were selected to indicate HRV in this study, the root mean square of successive differences between normal heartbeats (RMSSD) and the percentage of adjacent NN intervals that differ from each other by more than 50 ms (pNN50). Frequency-domain analysis was not applicable in present study due to the ultra-short duration of the task trial. However, RMSSD and pNN50 were reported to be correlated with the high-frequency power, which reflects the parasympathetic activity. The pNN50 is especially sensitive to changes in short-term periods (Shaffer and Ginsberg, 2017).

### 2.3 Statistical analysis

SPSS version 24.0 for Windows (SPSS Inc., Chicago, IL, United States) and MATLAB (2022b, Mathworks, Natick, MA, United States) with customized scripts were used for statistical analysis. The Kolmogorov–Smirnov test was used to determine whether a certain dataset was normally distributed. One-way ANOVA with Tukey *post-hoc* analysis was used for continuous variables with normal distribution, including SCWT-c, SCWT-ic, CFT, RRI. Chi-square test was applied for categorical variables. Kruskal–Wallis test and Mann–Whitney U test were used for analyzing non-normally distributed data, including MMSE, MoCA, TMT, DST, RMSSD, and pNN50. The significant level was set at *p* < 0.05.

## 3 Results

Ninety-three participants enrolled in the study. Among them, 31 participants in young adult group (YA), 44 in older adult group (OA), and 28 in older adults with MCI group (OA-MCI). Table 1 provides the basic information of the participants in three groups. Although the education level differed between young and older adults, the results remained unchanged after adjusting educational years with ANCOVA.

**TABLE 1:** Baseline characteristics, cognitive functions, executive functions in young adults, older adults and older adults with MCI.

	Young adults (*n* = 31)	Older adults (*n* = 44)	Older adults–MCI (*n* = 28)
Gender (M/F)	12/19	6/38	8/20
Age (y)	26.7 ± 2.1	70.9 ± 3.8^a^	73.0 ± 3.5^a^
Education (y)	17.6 ± 0.8	13.7 ± 2.7^a^	12.1 ± 3.5^a^
Height (cm)	166.9 ± 7.7	159.6 ± 7.3	158.5 ± 11.0
Weight (kg)	61.4 ± 10.3	59.2 ± 9.8	58.9 ± 11.5
History of hypertension (person)	0	42	28
History of diabetes (person)	0	35	35
MMSE	29.6 ± 0.8	28.5 ± 1.4^a^	27.2 ± 1.6^a^^,^^b^
MoCA	28.4 ± 1.4	27.8 ± 1.5	23.6 ± 1.5^a^^,^^b^
TMT-A (sec)	20.3 ± 6.9	37.1 ± 12.8^a^	49.2 ± 22.1^a^^,^^b^
TMT-B (sec)	39.6 ± 18.3	70.2 ± 28.0^a^	97.6 ± 33.1^a^^,^^b^
DST Score	23.6 ± 3.5	19.2 ± 3.6^a^	15.5 ± 3.5^a^^,^^b^

The values are shown as mean ± SD. MMSE, mini-mental status examination; MoCA, Montreal cognitive assessment; TMT, Trail Making Test.

^a^*p* < 0.05 compared to young adults, by one-way ANOVA with *post-hoc* Tukey test.

^b^*p* < 0.05 compared to older adults, by one-way ANOVA with *post-hoc* Tukey test.

### 3.1 Global cognitive function and executive functions

Table 1 shows the global cognitive and executive functions. YA had the highest score in global cognitive functions as indicated by MMSE, and EF as indicated by TMT-A, TMT-B, and DST. OA showed lower scores in the aforementioned outcomes compared to YA, yet higher compared to OA-MCI. However, MoCA only showed significant difference between OA and OA-MCI.

### 3.2 Performances in the cognitive task paradigm

The results of the cognitive performance during executing the task paradigm were displayed in Table 2. As comparing YA and OA, significant differences were found in SCWT-c (*p* = 0.002) and SCWT-ic (*p* < 0.001), but not CFT (*p* = 0.219). As comparing OA and OA-MCI, significant differences were detected in SCWT-ic (*p* = 0.012) and CFT (*p* = 0.040), but not SCWT-c (*p* = 0.094).

**TABLE 2:** The cognitive task performances in young adults, older adults and older adults with MCI.

	Young adults (*n* = 31)	Older adults (*n* = 44)	Older adults–MCI (*n* = 28)	*P*-value for ANOVA
SCWT-c (word)	140.0 ± 25.0	113.8 ± 22.5^a^	101.4 ± 18.7^a^	0.001
SCWT-ic (word)	76.5 ± 10.8	58.0 ± 11.7^a^	50.3 ± 8.5^a^^,^^b^	<0.001
CFT (item)	22.0 ± 3.9	18.3 ± 3.8	15.4 ± 4.0^a^^,^^b^	0.046

The values are shown as mean ± SD. SCWT-c, Stroop color word test with congruent; SCWT-ic, Stroop color word test with congruent; CFT, category fluency test.

^a^*p* < 0.05 compared to young adults, by one-way ANOVA with *post-hoc* Tukey test.

^b^*p* < 0.05 compared to older adults, by one-way ANOVA with *post-hoc* Tukey test.

### 3.3 Prefrontal activity

**FIGURE 3:**
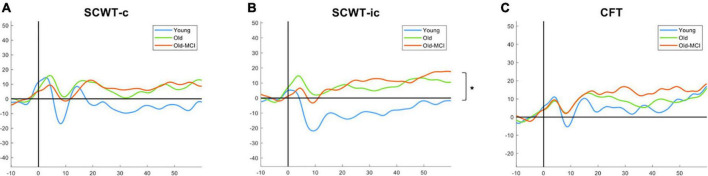
The HbO among young adults (blue), older adults (green), and older adults with MCI (orange) in SCWT-c **(A)**, SCWT-ic **(B)**, and CFT **(C)**. SCWT-c and -ic = Stroop color-word test in congruent and incongruent condition. *The asterisk symbol indicates significant difference between young adults and older adults by one-way ANOVA with *post-hoc* Tukey test at *p* < 0.05.

Figure 3 presents the PFC changes of HbO during three different tasks in 3 groups. We only found statistical significance between YA and OA (*p* = 0.007) during SCWT-ic but not during other 2 tasks. There was no significant difference between OA group and OA-MCI group in any of the 3 tasks tested.

### 3.4 Cardiac metrics and HRV

Table 3 shows the HRV results in three groups in resting and during three different tasks. RRI did not show any significant difference between groups during resting or during any tasking. For the RMSSD, there were significant differences between YA and OA during resting (*p* = 0.014), SCWT-c (*p* = 0.017), SCWT-ic (*p* = 0.002), and CFT (*p* = 0.006). For the pNN50, significant differences were also found between YA and OA during resting (*p* = 0.01), SCWT-c (*p* = 0.002), SCWT-ic (*p* = 0.001), and CFT (*p* = 0.002). However, no group differences were detected between OA and OA-MCI.

**TABLE 3:** Heart rate variability parameters in resting state and during task execution.

	Young adults (*n* = 31)	Older adults (*n* = 44)	Older adults–MCI (*n* = 28)	*P*-value for ANOVA
**RRI (ms)**				
Resting	802.1 ± 129.4	830.2 ± 133.2	823.9 ± 90.6	0.659
SCWT-c	742.1 ± 127.4	795.2 ± 138.0	790.4 ± 87.3	0.223
SCWT-ic	745.0 ± 132.9	793.4 ± 136.4	796.0 ± 87.5	0.268
CFT	744.0 ± 142.4	801.9 ± 135.1	791.5 ± 76.7	0.196
**RMSSD (ms)**				
Resting	37.7 ± 16.8	28.9 ± 20.6^a^	23.3 ± 17.5^a^	0.049
SCWT-c	32.6 ± 16.2	24.4 ± 18.2^a^	18.7 ± 16.1^a^	0.039
SCWT-ic	34.6 ± 17.5	22.1 ± 16.9^a^	19.4 ± 16.7^a^	0.005
CFT	29.8 ± 13.9	23.1 ± 18.3^a^	21.0 ± 17.9^a^	0.029
**pNN50 (%)**				
Resting	18.4 ± 14.2	8.3 ± 12.4^a^	3.8 ± 7.0^a^	0.001
SCWT-c	14.4 ± 13.4	6.6 ± 11.5^a^	4.2 ± 9.0^a^	0.011
SCWT-ic	14.9 ± 13.3	5.5 ± 10.8^a^	3.8 ± 9.2^a^	0.002
CFT	12.6 ± 12.2	5.8 ± 10.5^a^	3.9 ± 8.9^a^	0.019

The values are shown as mean ± SD. RRI, R-R interval; SCWT-c, Stroop color word test with congruent; SCWT-ic, Stroop color word test with congruent; CFT, category fluency test; RMSSD, root mean square of the successive differences; pNN50, proportion of NN50 divided by the total number of NN (R-R) intervals. NN50 is the number of times successive heartbeat intervals exceed 50 ms.

^a^*p* < 0.05 compared to young adults, by one-way ANOVA with *post-hoc* Tukey test.

However, the RRI, indicating heart rate, did not seem to change in resting or tasking in any of the three groups. We thus took a step further and analyzed the change in RRI from resting to task execution. After averaging the change values across three tasks within each group, YA demonstrated greater RRI change (58.4 ± 21.1 ms) than OA (33.3 ± 9.5 ms, *p* = 0.002) and OA-MCI (18.7 ± 18.3 ms, *p* = 0.017).

## 4 Discussion

This study demonstrated the differences of global cognitive functions, executive functions, heart rate variability, and increase in prefrontal activity in YA, OA, and OA-MCI. Interestingly, we found that YA had best performance in the assessments of cognitive and executive functions, followed by OA, and OA-MCI had the poorest performance among three groups. However, PFC activity and HRV only showed differences between YA and OA. There were no significant differences detected between OA and OA-MCI.

In this study, we noted that EF and cognitive functions showed a decrease between different age groups (YA vs. OA), whereas there was an increase in the activation of PFC during SCWT-ic. The SCWT-ic measures the ability to inhibit cognitive interference in addition to attention, processing speed, cognitive flexibility, and working memory. These age-related findings during SCWT-ic in PFC may indicate the possible neural compensatory mechanisms (Yap et al., 2017; Udina et al., 2020), or compensation-related utilization of neural circuits hypothesis (CRUNCH) for relatively more challenging tasks (Reuter-Lorenz and Cappell, 2008). When the cognitive demand increases, more cortical resources are activated. High task complexity, such as SCWT-ic, may challenge the brain for compensation-related utilization in OA. However, despite the compensatory activation, the task performance in OA was still significantly lower as compared to YA. We further noted such compensation-related utilization was not significant during SCWT-c or CFT, which was in line with Yoon et al.’s (2019) study. In Yoon et al.’s (2019) study, they showed SCWT-ic was a more suggestive of neural compensatory mechanisms than CFT.

The PFC activations did not differ significantly in any of the three tasks between OA and OA-MCI, but the task performances were worse especially the SCWT-ic and CFT in OA-MCI as compared with OA. Previously, we also noted no significant difference in PFC during usual walking and cognitive dual task walking between OA and OA-MCI (Weng et al., 2023). We thus speculate that the inability to activate more PFC for compensation may lead to poorer performance, both cognition and motor, in people with MCI as compared with healthy older adults.

The HRV, on the other hand, acts as the modulated consequence of the central-peripheral neural feedback. HRV could be seen as the responsiveness to the executive task demands (Thayer et al., 2009; Mathewson et al., 2010; Luque-Casado et al., 2016). In present study, HRV was observed from three aspects, RRI, RMSSD, and pNN50 during resting and task execution. Similar to PFC activations, RMSSD and pNN50 showed significant differences between two age groups (YA vs. OA) but not between OA and OA-MCI. Previous studies have presented performance-related reduction in RRI as the increased autonomic responsiveness in cognitive and EF task paradigm (Hansen et al., 2003; Mathewson et al., 2010). Faster heart rate usually reacts in parallel with lower baroreflex sensitivity. The greater autonomic reactivity including increased task-related heart rate and reduced vagal tone, was believed to potentially predict the EF performance. Age would weaken the autonomic control and EF performance (Mathewson et al., 2010), which was consistent with our findings.

The significant decrease in RMSSD in resting and during task in OA as compared to YA, further supports the age-related decrease in parasympathetic activity. Previous studies also showed the close relationships between decreased RMSSD and decreased executive function in older adults (de Vilhena Toledo and Junqueira, 2010; Eggenberger et al., 2020). Although the ECG recording for each task trial lasted for only one minute in present study, it was still possible to detect the differences between YA and OA. Potentially, RMSSD can be a HRV variable used in ultra-short recording period (Esco and Flatt, 2014; Laborde et al., 2017). The pNN50, another commonly used index for parasympathetic activity, also highly correlates with RMSSD (Umetani et al., 1998; Shaffer and Ginsberg, 2017). In our findings, OA had significantly lower pNN50 in all three tasks compared to YA, strengthening the results of attenuated HRV.

Recently, an increasing number of studies have addressed the associations between HRV and cognitive performances. In general, lower HRV was associated with poorer global cognition and performance in multiple cognitive domains, in which EF was the most investigated (Forte et al., 2019). Most studies measure resting HRV while some used reactive or task-induced HRV, however, with very different protocols. Nicolini et al. (2014) showed that older people with MCI exhibited small HRV changes to postural provocation compared to healthy older adults, even though there were no significant differences between two groups in resting. Marshall et al. (2018) showed that older people with frontotemporal dementia had degraded ability to identify facial emotion, alongside with attenuated cardiac reactivity. McDermott et al. (2019) showed that although older people with amnestic MCI had similar level of stress regulation as healthy older people, there was a positive correlation between pressure regulation index and HRV yet with no significant group effect. Lin et al. (2016) reported an association between lower mental fatigability and greater rebound in reactive HRV in people with MCI. Although the types of stimuli were not directly comparable to our study protocol, these previous studies suggested that older people with MCI were likely to have decreased cardiac reactivity in association with poorer performances, which was not consistent with our funding in the current study. Grässler et al. (2023) reported the significant decreases in RMSSD from resting to a Stroop task in people with MCI as compared with healthy control, indicating the vagal withdrawal in people with MCI. However, our results did not show the significant effects of cognitive impairment on autonomic control. It was worth noting that most studies that detected the difference between healthy and people with MCI acquired ECG recording in longer periods. Despite the fact that longer recording periods provides better validity in accuracy and predictive power, short and ultra-short periods provide greater possibility in clinical application whereas the usage of ultra-short term, as the proxies of long- and short-term, needs more research (Shaffer et al., 2020). As we explored the ultra-short time period during task execution, we did not find statistical significance impacted by mild cognitive impairment.

There are several limitations in this study. Due to the limited time period in ECG recordings, we were not able to process frequency-domain analysis. However, the indices chosen in this study still show significant findings between different age groups, which are in line with previous studies. We believe that short-term epochs as in our protocols were possible for detecting cognitive task-induced changes between younger and older adults, but unclear for MCI. The results in this study should be interpreted with care because it was a cross-sectional study and the associations of the outcomes did not indicate causality. Replication in larger cohorts with more evenly distributed gender and participants are needed.

## 5 Conclusion

Cognitive decline was seen in older adults, and even larger in people with MCI. Older people showed lower performances in general cognitive function and executive function, combined with compensatory increase of PFC activity, and reduced HRV. However, the PFC activity and HRV reaction was similar in healthy older adults and those with mild cognitive impairment.

## Data availability statement

The data that support the findings of this study are available from the corresponding author, upon reasonable request.

## Ethics statement

The studies involving humans were approved by the Institutional Review Board of National Yang Ming Chiao Tung University. The studies were conducted in accordance with the local legislation and institutional requirements. The participants provided their written informed consent to participate in this study.

## Author contributions

P-HK: Conceptualization, Investigation, Methodology, Visualization, Writing – original draft, Writing – review & editing. Y-RY: Methodology, Writing – review & editing. N-CY: Formal analysis, Software, Writing – original draft. P-YL: Investigation, Project administration, Writing – original draft. C-FL: Resources, Software, Writing – review & editing. R-YW: Conceptualization, Funding acquisition, Supervision, Writing – review & editing.

## References

[B1] Benarroch E. E. (1993). The central autonomic network: Functional organization, dysfunction, and perspective. *Mayo Clin. Proc.* 68 988–1001. 8412366 10.1016/s0025-6196(12)62272-1

[B2] Clément F. Gauthier S. Belleville S. (2013). Executive functions in mild cognitive impairment: Emergence and breakdown of neural plasticity. *Cortex* 49 1268–1279. 22841389 10.1016/j.cortex.2012.06.004

[B3] Collins O. Dillon S. Finucane C. Lawlor B. Kenny R. A. (2012). Parasympathetic autonomic dysfunction is common in mild cognitive impairment. *Neurobiol. Aging* 33 2324–2333. 22188719 10.1016/j.neurobiolaging.2011.11.017

[B4] da Silva V. P. Ramalho Oliveira B. R. Tavares Mello R. G. Moraes H. Deslandes A. C. Laks J. (2018). Heart rate variability indexes in dementia: A systematic review with a quantitative analysis. *Curr. Alzheimer Res.* 15 80–88. 10.2174/1567205014666170531082352 28558638

[B5] Daulatzai M. A. (2012). Dysfunctional nucleus tractus solitarius: Its crucial role in promoting neuropathogentic cascade of Alzheimer’s dementia–a novel hypothesis. *Neurochem. Res.* 37 846–868. 10.1007/s11064-011-0680-2 22219130

[B6] Davis S. W. Dennis N. A. Buchler N. G. White L. E. Madden D. J. Cabeza R. (2009). Assessing the effects of age on long white matter tracts using diffusion tensor tractography. *Neuroimage* 46 530–541. 10.1016/j.neuroimage.2009.01.068 19385018 PMC2775533

[B7] de Vilhena Toledo M. A. Junqueira L. F. (2010). Cardiac autonomic modulation and cognitive status in Alzheimer’s disease. *Clin. Autonom. Res.* 20 11–17. 10.1007/s10286-009-0035-0 19830511

[B8] Deary I. Bastin M. Pattie A. (2006). White matter integrity and cognition in childhood and old age. *Neurology* 66 505–512. 16505302 10.1212/01.wnl.0000199954.81900.e2

[B9] Eggenberger P. Annaheim S. Kündig K. A. Rossi R. M. Münzer T. de Bruin E. D. (2020). Heart rate variability mainly relates to cognitive executive functions and improves through exergame training in older adults: A secondary analysis of a 6-month randomized controlled trial. *Front. Aging Neurosci.* 12:197. 10.3389/fnagi.2020.00197 32760267 PMC7373948

[B10] Emery L. Heaven T. J. Paxton J. L. Braver T. S. (2008). Age-related changes in neural activity during performance matched working memory manipulation. *Neuroimage* 42 1577–1586. 18634891 10.1016/j.neuroimage.2008.06.021 PMC3192445

[B11] Esco M. R. Flatt A. A. (2014). Ultra-short-term heart rate variability indexes at rest and post-exercise in athletes: Evaluating the agreement with accepted recommendations. *J. Sports Sci. Med.* 13:535. 25177179 PMC4126289

[B12] Forte G. Favieri F. Casagrande M. (2019). Heart rate variability and cognitive function: A systematic review. *Front. Neurosci.* 13:710. 10.3389/fnins.2019.00710 31354419 PMC6637318

[B13] Funahashi S. Andreau J. M. (2013). Prefrontal cortex and neural mechanisms of executive function. *J. Physiol. Paris* 107 471–482. 23684970 10.1016/j.jphysparis.2013.05.001

[B14] Gianaros P. J. Van der Veen F. M. Jennings J. R. (2004). Regional cerebral blood flow correlates with heart period and high-frequency heart period variability during working-memory tasks: Implications for the cortical and subcortical regulation of cardiac autonomic activity. *Psychophysiology* 41 521–530. 10.1111/1469-8986.2004.00179.x 15189475 PMC4301264

[B15] Grässler B. Dordevic M. Darius S. (2023). Is there a link between heart rate variability and cognitive decline? A cross-sectional study on patients with mild cognitive impairment and cognitively healthy controls. *Arq. Neuro Psiquiatria* 81 9–18. 10.1055/s-0042-1758862 36918002 PMC10014205

[B16] Hansen A. L. Johnsen B. H. Thayer J. F. (2003). Vagal influence on working memory and attention. *Int. J. Psychophysiol.* 48 263–274. 12798986 10.1016/s0167-8760(03)00073-4

[B17] Heuninckx S. Wenderoth N. Swinnen S. P. (2008). Systems neuroplasticity in the aging brain: Recruiting additional neural resources for successful motor performance in elderly persons. *J. Neurosci.* 28 91–99. 10.1523/JNEUROSCI.3300-07.2008 18171926 PMC6671150

[B18] Heuninckx S. Wenderoth N. Debaere F. Peeters R. Swinnen S. P. (2005). Neural basis of aging: The penetration of cognition into action control. *J. Neurosci.* 25 6787–6796. 10.1523/JNEUROSCI.1263-05.2005 16033888 PMC6725362

[B19] Holtzer R. Izzetoglu M. (2020). Mild cognitive impairments attenuate prefrontal cortex activations during walking in older adults. *Brain Sci.* 10:415. 10.3390/brainsci10070415 32630216 PMC7407944

[B20] Johnsen B. H. Thayer J. F. Laberg J. C. (2003). Attentional and physiological characteristics of patients with dental anxiety. *J. Anxiety Disord.* 17 75–87. 12464290 10.1016/s0887-6185(02)00178-0

[B21] Laborde S. Mosley E. Thayer J. F. (2017). Heart rate variability and cardiac vagal tone in psychophysiological research–recommendations for experiment planning, data analysis, and data reporting. *Front. Psychol.* 8:213. 10.3389/fpsyg.2017.00213 28265249 PMC5316555

[B22] Liang P. Wang Z. Yang Y. Jia X. Li K. (2011). Functional disconnection and compensation in mild cognitive impairment: Evidence from DLPFC connectivity using resting-state fMRI. *PLoS One* 6:e22153. 10.1371/journal.pone.0022153 21811568 PMC3141010

[B23] Lin F. Ren P. Cotton K. Porsteinsson A. Mapstone M. Heffner K. L. (2016). Mental fatigability and heart rate variability in mild cognitive impairment. *Am. J. Geriatr. Psychiatry* 24 374–378. 10.1016/j.jagp.2015.12.012 26905050 PMC4846469

[B24] Luque-Casado A. Perales J. C. Cárdenas D. Sanabria D. (2016). Heart rate variability and cognitive processing: The autonomic response to task demands. *Biol. Psychol.* 113 83–90. 26638762 10.1016/j.biopsycho.2015.11.013

[B25] Marshall C. R. Hardy C. J. Allen M. (2018). Cardiac responses to viewing facial emotion differentiate frontotemporal dementias. *Ann. Clin. Transl. Neurol.* 5 687–696. 29928652 10.1002/acn3.563 PMC5989744

[B26] Mathewson K. J. Jetha M. K. Drmic I. E. (2010). Autonomic predictors of Stroop performance in young and middle-aged adults. *Int. J. Psychophysiol.* 76 123–129. 10.1016/j.ijpsycho.2010.02.007 20193717

[B27] McDermott K. Ren P. Lin F. (2019). The mediating role of hippocampal networks on stress regulation in amnestic mild cognitive impairment. *Neurobiol. Stress* 10:100162. 10.1016/j.ynstr.2019.100162 31193516 PMC6535625

[B28] Nicolini P. Ciulla M. M. Malfatto G. Abbate C. Mari D. Rossi P. D. (2014). Autonomic dysfunction in mild cognitive impairment: Evidence from power spectral analysis of heart rate variability in a cross-sectional case-control study. *PLoS One* 9:e96656. 10.1371/journal.pone.0096656 24801520 PMC4011966

[B29] Nikolin S. Boonstra T. W. Loo C. K. Martin D. (2017). Combined effect of prefrontal transcranial direct current stimulation and a working memory task on heart rate variability. *PLoS One* 12:e0181833. 10.1371/journal.pone.0181833 28771509 PMC5542548

[B30] Petersen R. C. Caracciolo B. Brayne C. Gauthier S. Jelic V. Fratiglioni L. (2014). Mild cognitive impairment: A concept in evolution. *J. Intern. Med.* 275 214–228. 24605806 10.1111/joim.12190 PMC3967548

[B31] Reuter-Lorenz P. A. Cappell K. A. (2008). Neurocognitive aging and the compensation hypothesis. *Curr. Dir. Psychol. Sci.* 17 177–182.

[B32] Ruchinskas R. (2019). Wechsler adult intelligence scale-digit span performance in subjective cognitive complaints, amnestic mild cognitive impairment, and probable dementia of the Alzheimer type. *Clin. Neuropsychol.* 33 1436–1444. 10.1080/13854046.2019.1585574 30931811

[B33] Saint Martin M. Sforza E. Thomas-Anterion C. Barthélémy J. C. Roche F. (2013). Baroreflex sensitivity, vascular risk factors, and cognitive function in a healthy elderly population: The PROOF cohort. *J. Am. Geriatr. Soc.* 61 2096–2102. 10.1111/jgs.12548 24279643

[B34] Salat D. H. Kaye J. A. Janowsky J. S. (2001). Selective preservation and degeneration within the prefrontal cortex in aging and Alzheimer disease. *Arch. Neurol.* 58 1403–1408. 10.1001/archneur.58.9.1403 11559311

[B35] Sánchez-Cubillo I. Periáñez J. A. Adrover-Roig D. Rodríguez-Sánchez J. M. Ríos-Lago M. Tirapu J. (2009). Construct validity of the trail making test: Role of task-switching, working memory, inhibition/interference control, and visuomotor abilities. *J. Int. Neuropsychol. Soc.* 15 438–450. 10.1017/S1355617709090626 19402930

[B36] Scarpina F. Tagini S. (2017). The stroop color and word test. *Front. Psychol.* 8:557. 10.3389/fpsyg.2017.00557 28446889 PMC5388755

[B37] Shaffer F. Ginsberg J. P. (2017). An overview of heart rate variability metrics and norms. *Front. Public Health* 5:258. 10.3389/fpubh.2017.00258 29034226 PMC5624990

[B38] Shaffer F. Meehan Z. M. Zerr C. L. (2020). A critical review of ultra-short-term heart rate variability norms research. *Front. Neurosci.* 14:594880. 10.3389/fnins.2020.594880 33328866 PMC7710683

[B39] Shah A. J. Su S. Veledar E. (2011). Is heart rate variability related to memory performance in middle aged men? *Psychosom. Med.* 73:475. 10.1097/PSY.0b013e3182227d6a PMC3307789 21715297

[B40] Shao Z. Janse E. Visser K. Meyer A. S. (2014). What do verbal fluency tasks measure? Predictors of verbal fluency performance in older adults. *Front. Psychol.* 5:772. 10.3389/fpsyg.2014.00772 25101034 PMC4106453

[B41] Sperling R. (2007). Functional MRI studies of associative encoding in normal aging, mild cognitive impairment, and Alzheimer’s disease. *Ann. N. Y. Acad. Sci.* 1097 146–155. 17413017 10.1196/annals.1379.009

[B42] Thayer J. F. Lane R. D. (2009). Claude Bernard and the heart–brain connection: Further elaboration of a model of neurovisceral integration. *Neurosci. Biobehav. Rev.* 33 81–88. 10.1016/j.neubiorev.2008.08.004 18771686

[B43] Thayer J. F. Hansen A. L. Saus-Rose E. Johnsen B. H. (2009). Heart rate variability, prefrontal neural function, and cognitive performance: The neurovisceral integration perspective on self-regulation, adaptation, and health. *Ann. Behav. Med.* 37 141–153. 10.1007/s12160-009-9101-z 19424767

[B44] Udina C. Avtzi S. Durduran T. Rosso A. L. Castellano-Tejedor C. Perez L. M. (2020). Functional near-infrared spectroscopy to study cerebral hemodynamics in older adults during cognitive and motor tasks: A review. *Front. Aging Neurosci.* 11:367. 10.3389/fnagi.2019.00367 32038224 PMC6985209

[B45] Umetani K. Singer D. H. McCraty R. Atkinson M. (1998). Twenty-four hour time domain heart rate variability and heart rate: Relations to age and gender over nine decades. *J. Am. Coll. Cardiol.* 31 593–601. 10.1016/s0735-1097(97)00554-8 9502641

[B46] Vasudev A. Saxby B. K. O’Brien J. T. Colloby S. J. Firbank M. J. Brooker H. (2012). Relationship between cognition, magnetic resonance white matter hyperintensities, and cardiovascular autonomic changes in late-life depression. *Am. J. Geriatr. Psychiatry* 20 691–699. 10.1097/JGP.0b013e31824c0435 22609766

[B47] Wang R. Y. Zhou J. H. Huang Y. C. Yang Y. R. (2018). Reliability of the Chinese version of the trail making test and Stroop color and word test among older adults. *Int. J. Gerontol.* 12 336–339.

[B48] Weng W.-H. Yang Y.-R. Yeh N.-C. Ku P. H. Wang P. S. Liao Y. Y. (2023). Gait performance and prefrontal cortex activation during single and dual task walking in older adults with different cognitive levels. *Front. Aging Neurosci.* 15:1177082. 10.3389/fnagi.2023.1177082 37333460 PMC10272571

[B49] Xu P. Chen A. Li Y. Xing X. Lu H. (2019). Medial prefrontal cortex in neurological diseases. *Physiol. Genom.* 51 432–442. 10.1152/physiolgenomics.00006.2019 PMC6766703 31373533

[B50] Yap K. H. Ung W. C. Ebenezer E. G. Nordin N. Chin P. S. Sugathan S. (2017). Visualizing hyperactivation in neurodegeneration based on prefrontal oxygenation: A comparative study of mild Alzheimer’s disease, mild cognitive impairment, and healthy controls. *Front. Aging Neurosci.* 9:287. 10.3389/fnagi.2017.00287 28919856 PMC5585736

[B51] Ye B. Seo S. Yang J. J. Kim H. J. Kim Y. J. Yoon C. W. (2014). Comparison of cortical thickness in patients with early-stage versus late-stage amnestic mild cognitive impairment. *Eur. J. Neurol.* 21 86–92. 10.1111/ene.12251 24033766

[B52] Yeung M. K. Chan A. S. (2020). Functional near-infrared spectroscopy reveals decreased resting oxygenation levels and task-related oxygenation changes in mild cognitive impairment and dementia: A systematic review. *J. Psychiatr. Res.* 124 58–76. 10.1016/j.jpsychires.2020.02.017 32120065

[B53] Yoon J. A. Kong I. J. Choi J. Baek J. Y. Kim E. J. Shin Y. I. (2019). Neural compensatory response during complex cognitive function tasks in mild cognitive impairment: A near-infrared spectroscopy study. *Neural Plast.* 2019:7845104. 10.1155/2019/7845104 31320893 PMC6607700

